# Recurrence after pituitary surgery in adult Cushing’s disease: a systematic review on diagnosis and treatment

**DOI:** 10.1007/s12020-020-02432-z

**Published:** 2020-08-02

**Authors:** Leah T. Braun, German Rubinstein, Stephanie Zopp, Frederick Vogel, Christine Schmid-Tannwald, Montserrat Pazos Escudero, Jürgen Honegger, Roland Ladurner, Martin Reincke

**Affiliations:** 1 Department of Endocrinology, Medizinische Klinik und Poliklinik IV, Klinikum der Universität München, München, Germany; 2 Klinik und Poliklinik für Radiologie, Klinikum der Universität München, München, Germany; 3 Klinik und Poliklinik für Strahlentherapie und Radioonkologie, Klinikum der Universität München, München, Germany; 4 Department for Neurosurgery, University Hospital Tübingen, 72076 Tübingen, Germany; 5 Klinik für Allgemeine, Unfall- und Wiederherstellungschirurgie, Campus Innenstadt, Klinikum der Universität München, München, Germany

**Keywords:** Hypercortisolism, Cushing’s syndrome, Pituitary adenoma, Adrenostatic therapy

## Abstract

**Purpose:**

Recurrence after pituitary surgery in Cushing’s disease (CD) is a common problem ranging from 5% (minimum) to 50% (maximum) after initially successful surgery, respectively. In this review, we give an overview of the current literature regarding prevalence, diagnosis, and therapeutic options of recurrent CD.

**Methods:**

We systematically screened the literature regarding recurrent and persistent Cushing’s disease using the MESH term *Cushing’s disease and recurrence*. Of 717 results in PubMed, all manuscripts in English and German published between 1980 and April 2020 were screened. Case reports, comments, publications focusing on pediatric CD or CD in veterinary disciplines or studies with very small sample size (patient number < 10) were excluded. Also, papers on CD in pregnancy were not included in this review.

**Results and conclusions:**

Because of the high incidence of recurrence in CD, annual clinical and biochemical follow-up is paramount. 50% of recurrences occur during the first 50 months after first surgery. In case of recurrence, treatment options include second surgery, pituitary radiation, targeted medical therapy to control hypercortisolism, and bilateral adrenalectomy. Success rates of all these treatment options vary between 25 (some of the medical therapy) and 100% (bilateral adrenalectomy). All treatment options have specific advantages, limitations, and side effects. Therefore, treatment decisions have to be individualized according to the specific needs of the patient.

## Introduction

Cushing’s syndrome (CS) is in 60–85% of cases ACTH-dependent. Most frequently it is caused by a corticotroph adenoma of the pituitary gland [1]. The recommended screening tests for hypercortisolism include the low-dose dexamethasone suppression test (LDDST with 1 mg), urinary free cortisol in a 24 h-output-collection (UFC), and midnight salivary cortisol measurement. The tests for subtyping include the high-dose-dexamethasone suppression test, the CRH test, and in some cases the inferior petrosal sampling. According to recent systematic meta-analyses, first-line therapy is transsphenoidal surgery which leads to remission in 71% [2], 80% [3] or 78% of cases [4]. However, there are still open questions regarding diagnosis and therapy of recurrent CD. To summarize the current research in this field and to identify knowledge gaps, we conducted a systematic review.

## Methods

We systematically screened the literature regarding recurrent and persistent Cushing’s disease (CD) using the MESH term *Cushing’s disease* and *recurrence* in PubMed. We identified 717 studies published between 1980 and April 2020, of which 692 were in English or German. Of these manuscripts, 134 were fully considered after excluding case reports, comments, reviews without meta-analysis, publications focusing on pediatric CD or CD in veterinary disciplines, CD in pregnancy and very small studies with sample size <10 patients. Table 1 summarizes major studies with ≥100 patients with CD published between 1983 and 2018 in which recurrence after first transsphenoidal surgery was analyzed.

**Table 1:** Recurrence and persistency rates in studies with *N* ≥ 100, divided into <5 years of follow-up and more than 5 years of follow-up (partly adapted from [4])

First author	Year of publication	*N*	Remission rate (%)	Definition of remission	Recurrence rate (%)	Definition of recurrence
*More than 5 years of follow-up*						
Cavagnini [94]	2001	288	70	–	15	–
Flitsch [95]	2003	147	93	Morning serum cortisol	5.6	Not defined
Hammer [96]	2004	289	82	Basal or dexamethasone-suppressed plasma cortisol level of 5 μg/dl or less in the first week after surgery	9	Initial remission followed by hypercortisolism or additional therapy after 6 months and more after TSS
Dimopoulou [97]	2013	120	71	UFC below or within normal range; serum cortisol below 5 µg/dl during LDDST	34	UFC elevated or lack of cortisol suppression during LDDST with clinical symptoms
Hofmann [57]	2008	426	75.9	Cortisol below 2 mg/dL after 2 mg-LDDST, 1 week or 3 months post-surgery	15	Pathological results in 2 mg-LDDST
Invitti [98]	1999	288	69	Signs of adrenal insufficiency, low or normal UFC, low or normal plasma morning ACTH and cortisol levels	17	Not exactly defined
Jagannathan [99]	2009	261	96.5	UFC below or in the normal range, or morning serum cortisol below 5 µg/dL	2.3	Clinical symptoms
Jehle [100]	2008	193	80.8	Normalized UFC, secondary adrenal insufficiency or serum cortisol below 1.8 µg/dL after LDDST	13.5	Recurrent hypercortisolism
Rollin [101]	2007	103	85.4 (first TSS) and 28.6 (second TSS)	Morning serum cortisol, LDDST	6.8	Not exactly defined
Sonino [102]	1996	103	76.7	Regression of Clinical signs, normal UFC, normal LDDST	25.9	Recurrence of both clinical and biochemical signs
Johnston [103]	2017	101	89 (microadenoma)	Adrenal insufficiency, normal UFC, late night salivary cortisol or LDDST	4–6	Not exactly defined
Chandler [104]	2016	276	89 (microadenoma), 66 (macroadenoma), 71 (negative imaging)	Adrenal insufficiency, low morning cortisol, normal UFC	17	Recurrence of symptoms and biochemical hypercortisolism
Alexandraki [105]	2013	131	72.8 (microadenoma), 42.9 (macroadenoma)	Adrenal insufficiency, serum cortisol <50 nmol/l	12 (microadenoma)	LDDST > 50 nmol/l and recurrence of clinical symptoms
Bansal [106]	2017	230	65.6	Adrenal insufficiency, normal LDDST	41	Serum cortisol > 1.8 µg/dL after LDDST
*Less than 5 years of follow-up*						
Bochicchio [7]	1995	668	76.3	LDDST	12.7	Clinical and biochemical recurrence
Boggan [107]	1983	100	92 (microadenoma), 45 (macroadenoma)	Regression of clinical symptoms, normal plasma ACTH and cortisol, normal LDDST	2 and 14, respectively	Not exactly defined
Bou Khalil [108]	2011	101	79.5	Normal morning cortisol, normal UFC and normal salivary cortisol	21	One abnormal test result (UFC, LDDST, or salivary cortisol)
Chen [109]	2003	174	74	Low morning cortisol levels	26	Elevated UFC, elevated serum cortisol, abnormal diurnal rhythm
Hofmann [110]	2006	100	75	Adrenal insufficiency, cortisol after 2 mf LDDST below 2 g/dL	4.8	Not exactly defined
Knappe [111]	1996	310	85.2	–	1	–
Nakane [112]	1987	100	92	Plasma cortisol	9	Not exactly defined
Patil [58]	2008	215	85.5 (61% in second TSS)	Adrenal insufficiency or normal UFC	17.4	Clinical symptoms, elevated UFC
Prevedello [113]	2008	167	88.6	Adrenal insufficiency, low cortisol, normal UFC, regression of symptoms	12.8	Not exactly defined
Valassi [114]	2010	620	70.5	Low morning serum cortisol, normal UFC	13	Morning serum cortisol, UFC, abnormal LDDST
Feng [115]	2018	341	78.9 (higher in first TSS and macroadenoma)	Serum cortisol below 5 ug/dL	2.4	Abnormal UFC or serum cortisol

*UFC* urinary free cortisol, *LDDST* low-dose-dexamethasone suppression-test

## Definition of recurrence and persistency

Remission following transsphenoidal surgery is most often defined by low morning cortisol levels (<1.8 µg/dl; 50 nmol/L) [5] and the requirement of glucocorticoid replacement therapy. Obviously, there may be patients who do not fulfill this cut-off but still enter remission. In contrast to disease persistence after transsphenoidal surgery, the definition of recurrence requires a phase of months to years of disease remission, which then is followed by re-appearance of CD. Remission criteria vary between studies (see Table 1), which is one possible explanation for different remission and recurrence rates in different studies. While remission criteria are not standardized, recurrence criteria are also not consistent throughout different studies. Most of the studies define recurrence by an elevated UFC or elevated serum cortisol—criteria, which are not the most sensitive and specific markers.

## Diagnosis of recurrence

### Prevalence of recurrence after pituitary surgery

CD recurs in ~14% of patients (5–21%) between 3 and 158 months (mean 51 months) [4]. Fifty percent of relapses occur during the first 15–50 months after initial surgery [6]. However, late recurrences after decades of remission are possible [7]. A regular follow-up is therefore mandatory and a consistent recommendation in several studies and guidelines [8–11]. Recurrence rates differ greatly between the studies, most likely due to varying definitions of remission and recurrence, and also due to different surgical approaches and length of follow-up [12]. The recurrence rate is higher with longer follow-up, as already stated in 1992 by Tahir and Sheeler and shown in Table 1 [13]. In addition, comparisons among studies is difficult since, for example, few patients with negative MRI at baseline are included in some series [14], a factor that influences success greatly [15]. According to our research, recurrence of CD is mostly defined by biochemical criteria, while clinical signs and symptoms are often not mentioned and, therefore, apparently not compulsory. This scenario creates a level of ambiguity since biochemical evidence of hypercortisolism is not per se specific and sensitive. Examples for the latter are mild recurrence or cyclic CS [16] and for the former physiological forms of hypercortisolism (i.e., in major depression), which can also be typical in the postoperative phase of CD.

According to a recent multicenter study by Geer et al. the clinical practice situation in the US shows that transsphenoidal surgery is in more than 50% of the cases initially unsuccessful [17]. This study was retrospective based on data from medical records from 230 patients. Mean follow-up was quite short with 3 years (median 1.9, range 0–27.5 years) and a lot of data were missing. For example, there were no MRI results available for 90 patients [17]. After initial surgery, only 91 patients were in remission and, at the end of the observation period, 110 patients (49.1%) achieved remission using additional treatment strategies. Remission was not achieved in the other 67 patients, data of 47 patients were missing. Summarized, results from this study should be evaluated with caution as outcome differs greatly from results of recent meta-analysis. However, it is a warning signal that surgical series from expert neurosurgery centers may not reflect real world scenarios, in which access to expert centers and optimized follow-up may be limited.

### Factors influencing recurrence

Many studies have focused on factors influencing the remission state of patients with CD (summary shown in Table 2). In a single-center study, remission rates in macroadenomas are higher than in microadenomas [18], opposite to the findings of a recent metanalysis [19] and most of the other studies. Experience of the surgeon influences outcome, morbidity, and mortality [4, 20]. In a multicenter, retrospective European study of 668 patients remission rates were associated with pre-surgical identification of the tumor by MRI, an observation also reported by Chee et al. [21]. It was also higher in patients with long-term glucocorticoid replacement therapy and those with low postoperative cortisol levels [7], whereas only a minority did not confirm the latter [22–24].

**Table 2:** Predictors for remission

Predictors for remission	Studies
• Identification of the tumor pre-surgery by MRI	*Bochicchio et al. 1995* [7]*, Chee et al. 2001* [21]
• No invasion of the sinus cavernosus by the adenoma	*Cannavo et al. 2003* [116]
• Low postoperative cortisol levels (below normal ranges or not measurable, <2 µg/dL)	*Bochicchio et al. 1995* [7]*, Liu et al. 2019* [25]*, Fleseriu et al. 2016* [34]*, Pieters et al.1989* [117]*, Lindsay et al. 2011* [118]*, Aranda et al. 2015* [119]*, Imaki et al. 2001* [120]*, Ironside et al. 2018* [121]*, Mayberg et al. 2018* [122]
• Low cortisol levels 6–12 weeks after surgery (<35 nmol/l)	*Toms et al. 1993* [123]
• Long-term replacement therapy required (>1 year), long term of hypocortisolism (>1 year)	*Bochicchio et al. 1995* [7]*, Bansal et al. 2017* [106]
• Low postoperative ACTH levels (mean 7.9 ng/L or mean 13 pg/ml, respectively), ACTH value <3.3 pmol/L postoperative	*Liu et al. 2019* [25]*, Kuo et al. 2017* [26]*, Abellan-Galiana et al. 2019* [124]
• Histological confirmation of adenoma	*Pouratian et al. 2007* [29]*, Serban et al. 2020* [125]
• Lower DHEA levels pre-surgery	*Kuo et al. 2017* [26]
• Lower ACTH levels pre-surgery (mean = 71 ng/L)	*Liu et al. 2019* [25]*, Kuo et al. 2017* [26]*, Selek et al. 2018* [126]
• Significant decrease of BMI post-surgery	*Kuo et al. 2017* [26]
• Experience of the surgeon/the center	*Barker et al. 2003* [20]*, Honegger et al. 2018* [55]*, Rees et al. 2002* [127]*, Serban et al. 2020* [125]
• Age (mean age 35 years; recurrence more often in younger age below 35 years)	*Liu et al. 2019* [25]
• No USP8 mutant coricotroph tumor	*Albani et al. 2018* [27]
• Short time to recovery from postoperative adrenal insufficiency	*Berr et al. 2015* [28]
• Postoperative cortisol response to desmopressin (delta < 193 nmol/l), low cortisol and ACTH peak after desmopressin, response to desmopressin after 6 months	*Romanholi et al. 2008* [128]*, Valero et al. 2004* [129]*, Vassiliadi et al.2016* [130]*, Losa et al. 2009* [131]*, L Marc’hadour et al. 2015* [132]*, Barbot et al. 2013* [133]*, Losa et al. 2001* [134]*, Cambos et al. 2020* [42]
• Post-surgery 11-deoxycortisol < 150 nmol/l after metyrapone-test	*van Aken et al. 1997* [135]
• Cortisol < 49 nmol/L in a 48 h suppression test with betamethasone 2 mg/day	*Uvelius et al. 2018* [136]

According to a study by Liu et al. using different machine learning algorithms, the most important predictors for recurrence were young age, postoperative serum cortisol, and postoperative ACTH, both measured in the first 7 days after surgery (cut-offs see Table 2) [25]. However, sensitivity and specificity using such an algorithm were quite low with 87% and 58%, respectively.

In a retrospective study with 41 patients with CD, higher ACTH levels pre-surgery were one predictor for recurrence, while lower DHEA levels pre-surgery and a larger decrease of BMI after treatment were factors that were associated with remission [26]. Furthermore, recurrence is also influenced by the presence of somatic USP8 mutations and is significantly more frequent in patients with USP8 mutant corticotroph tumors [27]. Duration of symptoms until first surgery does not influence remission rate [28]. Another matter of debate are patients in whom a corticotroph pituitary adenoma is not identified histologically. In a study by Pouratian, an adenoma could not be confirmed by pathology in 111 out of 490 cases. Of these patients, only 50% achieved remission compared with 88% of patients with histologically confirmed adenoma [29]. Furthermore, these patients had higher rates of early recurrence within the first months after surgery [29]. In another study focusing on the pathology, recurrence was associated with the lack of peritumoral Crooke’s change [30] but this finding was not confirmed in a larger study [31]. However, it should be kept in mind that rate of diagnostic errors (misdiagnoses) in pathology ranges between 3–9% [32] and, therefore, false-negative and false-positive results are not uncommon. In a meta-analysis by Roelfsema, only postoperative hormone levels remained a prognostic factor for remission—as shown in 20 studies—while other factors such as age, gender, tumor size, and invasion were unrelated to recurrence [2].

## Diagnosis of recurrence

### Surveillance of patients in remission: how often performing follow-up studies?

It is a widely accepted practice to recommend screening of patients with a history of CD on a regular basis. The rational, as outlined above, is the lack of a reliable prediction model as no single or combined clinical or biochemical parameter can exclude future recurrence with acceptable precision [33, 34]. Currently, there is no consensus recommendation on the intervals of clinical and/or biochemical follow-up. Ayala et al. recommend to screen patients in different intervals—3 months to annually—depending on morning serum cortisol levels measured 2–3 days post-surgery [9]. Fleseriu et al. recommend to evaluate patients clinically on an annual basis [34] and to conduct a biochemical screening when the patient has new evidence of a tumor in the MRI or worsening/onset of symptoms and comorbidities that might be related to CS [34]. In an expert statement, Geer et al. suggested a complex and detailed scheme, taking into account the time passed since pituitary surgery, the requirement for steroid replacement therapy, and previous clinical and biochemical evidence of remission [35]. Suggested test intervals vary between 2 and 6 months. The overarching premises of these quite different recommendations is patient safety and cost effectiveness, within the frame of different health care systems. An additional factor to be considered is that close surveillance of patients can have a negative impact on well-being, similar to cancer patients who are reminded every time at posttreatment staging on their cancer history. As the vast majority all of recurrences occur in the first 10 years after pituitary surgery as shown in the meta-analysis by Roelfsema [2], we recommend as an minimum compromise an annual screening of patients with CD in the first 10 years after surgery. A study by Psaras et al. with 33 patients with CD revealed that 84.5% are followed-up by endocrinologists and only 9.1% of patients are not under aftercare at all, which is all in all satisfying but leaves room for improvement [36].

### Are there data on clinical evidence of recurrence in CD?

While there are different studies focusing on the biochemical recurrence of CD, the clinical course has seldom been addressed by studies—and never as a sole parameter but always in combination with biochemical results [4]. Therefore, questions remain, i.e., whether there is a certain sequence of events in which signs and symptoms recur. Studies addressing these aspects might be of value regarding treatment and screening decisions. The lack of studies addressing this topic is surprising when considering that there is still a debate on how to define recurrence biochemically [37].

### Biochemical evidence for recurrence

Diagnosis of recurrence is comparable to the diagnostic process at first diagnosis. Biochemical screening consists of the LDDST, the late-night salivary cortisol and the cortisol in a 24 h collection (UFC). In addition, a distinct increase of plasma ACTH can be helpful to further support the presence of recurrence. Of these tests, midnight salivary cortisol is the first test to become abnormal [38, 39], followed by LDDST, while UFC appears to be least sensitive [12], most likely because of the use of immunoassays with inappropriately wide normal ranges and unspecified cross-reactivity of the primary antibody with other steroids. However, in a study with 38 patients by Castinetti, a combined dexamethasone desmopressin test was an early marker for recurrence before other tests showed abnormal results [40]. The test was conducted 6 and 12 months after surgery and after that yearly. Similarly, Ambrogio et al. showed that the desmopressin test might be helpful to detect recurrence early [41]. In this study, desmopressin test was performed immediately after surgery (<1 week) and in the follow-ups (after a few months and yearly). A recent study reported that a cortisol response during the desmopressin test, performed in the first 3 month of the postoperative phase of hypocortisolism, might be quite predictive [42].

In a study by Atkinson et al., a significant number of patients had recurrence with cyclic hypercortisolism, suggesting that repetitive measurements of urine and salivary samples are crucial [6]. Further diagnostic subtyping, such as CRH stimulation test, the high-dose dexamethasone suppression test, or inferior sinus petrosal sampling is per se not required because the origin of hypercortisolism, based on positive histopathology and/or postoperative tertiary adrenal insufficiency, is obvious. Exceptions may be the rare case of cyclic CS of unknown origin in which the switch to ‘off’ phase’ coincided with the time of pituitary surgery, causing a diagnostic riddle.

### Imaging to detect recurrence

Failure to identify the tumor pre-surgery by MRI is associated with a higher rate of persistence and recurrence in several studies [7, 21]. A similar scenario should apply to patients with negative MRI at recurrence although no study has reported MRI positivity rates in the recurrence situation. It can be assumed that the adenoma should be located on the same side as initially [43]; in a study by Hofmann et al., the recurrent adenoma was always located on the same side as initially [44]. An inferior petrosal sampling can be helpful [15] to identify the location of the coricotroph adenoma (right or left)—at least in 70% of cases [45] in the setting of first surgery. However, there is no consensus whether to use inferior petrosal sampling solely for this purpose, and postoperative changes in venous anatomy may further reduce its value.

In a review by Vitale et al. the authors recommended to use three Tesla MRI in patients with negative standard 1.5 Tesla MRI and to use an optimized MRI protocol [46]. In a recent study with 23 patients with CD and a negative standard MRI, the combination of post-contrast FLAIR sequences and post-contrast 3D-GRE sequences was helpful in otherwise pre-operative undetectable microadenomas [47], as all five adenomas invisible by standard MRI were detectable by post-contrast FLAIR sequences. Grober et al. conducted a study with different MRI techniques to detect microadenoma, showing that a spoiled-gradient echo 3D T1 sequence has a higher sensitivity than dynamic contrast-enhanced MRIs and conventional MRI in surgery-naïve patients [48].

New imaging technologies such as the combination of MRI and PET were recently studied in 35 patients with therapy- naïve CD. While diagnostic accuracy using MRI was only 40%, the adenoma could be detected with the combination of methionine-PET/3.0 Tesla MRI in 100% of cases and with FDG-PET/3.0 Tesla MRI in 73% of cases [49]. The value of methionine PET-imaging to detect adenomas was also shown in another study with smaller sample size [50]. As shown in a different study, simultaneously CRH stimulation may improve the detection rate of adenomas by FDG-PET [51]. Unfortunately, there are no studies exclusively focusing on patients with recurrent disease.

## Treatment options after recurrence and in persistent Cushing’s syndrome

### Persistency versus recurrence

**Fig. 1:**
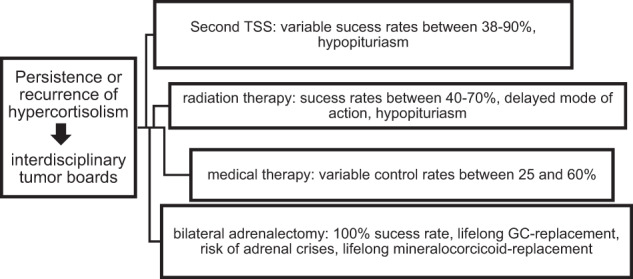
Treatment options after recurrence [93]

Treatment options in both scenarios are comparable with only little differences (overview: Fig. 1, Table 3). In persistent disease, it might be necessary to repeat or add diagnostic tests such as the inferior sinus petrosus sampling or to conduct further imaging to exclude an ectopic CS definitely. Furthermore, while a second pituitary surgery is normally the first treatment option in recurrent CD, it might not be in persistent CD with invasive growth of the adenoma. In these cases, when a complete removal of the tumor by surgery is unlikely, radiation therapy might be a more preferable option.

**Table 3:** Advantages and disadvantages of different treatment options

Therapy	Advantages	Disadvantages	Success rates
Second or third pituitary surgery	• Safe to perform when conducted by an experienced surgeon	• Hypopituriarism • Recurrence possible • Very variable success rates • Risk of surgery	Mean 64% (38–90%)
Radiation therapy	• Can be performed in patients that are not suitable candidates for surgery	• Hypopituitarism • Recurrence possible • Delayed mode of action; combination with medical therapy mandatory • Several treatment sessions in unfractionated radiation therapy	40–70% [137]
Medical therapy	• Can be performed in patients that are not suitable candidates for surgery • Bridge-therapy • Acute onset	• Side effects • Escape possible • High costs over the long term	25–60% (dependent on drug) [137], up to 82% for mitotane [75]
Bilateral adrenalectomy	• Definitive therapy • 100% success rate	• Life-long adrenal insufficiency, patients are at risk of addison’s crisis • Risk of Nelson Tumor • Risk of surgery	100%

### Second pituitary surgery (TSS)

The value of a second pituitary surgery was already shown in 1989 by Friedman et al. [52]. It has to be differentiated between a second TSS performed shortly after first TSS in persistent disease and a second TSS in recurrent disease. In persistent disease, the surgery is usually performed early (days to 4 weeks) before scar tissue has been developing [53]. Remission rate in persistent disease is about 54% while remission rates are higher (64%) in recurrent disease. A thorough review of outcomes in persistent versus recurrent CD can be found in Rubinstein et al. [54]. Pituitary surgery should be conducted in a center with high experience (more than 25 surgeries per year [55])—this applies both for persistent, recurrent, and surgery-naïve CD as well. A second pituitary surgery seems to be currently the first-line therapy in recurrent CD with a mean remission rate of 64%, but high rates of variability (38–90%) is reported in the literature [54]. Success rates seem to be lower than in first surgery [56]. Second TSS has more complication due to scar tissue in the pituitary. The risk of hypopituitarism is higher due to a more aggressive surgery [57], with incidences between 19 [58] and 64% [59].

### Radiation therapy

Radiation therapy can be conducted as conventional radiotherapy (fractionized over 25–30 sessions) or as stereotactic (unfractionated, single-dose) radiosurgery. In case of a negative MRI scan, radiosurgery should be delivered to the area of the adenoma at time of first diagnosis, while radiotherapy is delivered in this scenario to the region of the whole pituitary.

Radiotherapy is a valid therapeutic option, with median remission rates of 80% (123 patients in 7 studies), no recurrence rate and a median time to remission of 8 months [60]. However, cabergoline must not be used during radiotherapy, as it may increases recurrence rate due to a recently published study [61]. New hypopituitarism is a common problem in up to 38 [62]–40% of patients [63]. Other severe side effects are rare (optic neuropathy, radiation induced second tumors) [64] and the 10 year survival rate was very high with 95% in one study [64]. Response to radiotherapy does not correlate with sex, age, or severity of disease [65].

Stereotactic radiosurgery was introduced in Sweden in 1969 by Lars Leksell but was not widespread used to treat ACTH-producing adenomas in other countries until the 1980s [66]. Success of this treatment options depends on the size and location of the tumor. Obviously, best results are achieved in well-defined tumors [67]. Published remission rates are slightly lower than for fractionized radiotherapy: In a retrospective study with 68 patients, gamma knife radiosurgery turned out to be quite effective, leading to remission in 76% of patients within the next 5 years. Escape rate following radiosurgery was 13%, and 23% of patients suffered from new pituitary deficiencies [68]. Another study with 43 patients reported a remission rate of 63% [69]. A higher escape rate of 18% was reported in an international, multicenter study with 278 patients [70]. Forty-two percent of patients developed new hypopituitarism when stereotactic proton radiosurgery was used [71]. Remission rate using Cyberknife were lower in one study, reporting a remission rate of 57%, but the study population was very small (seven patients) and patients had CD with sinus cavernosus invasion [72]. Severe side effects with transient visual loss and permanent diplopia were seen in 2 out of 20 patients in another study [73]. Importantly, experience of the center is associated with remission rate [74].

The main disadvantage of radiation therapy is the relatively long time to remission. A combination with medical therapy is always required to bridge the time between radiation and remission. Effective medical control should be proven before initiation of radiation therapy. Continuous endocrine surveillance is mandatory to detect hypopituitarism early and also to adjust medical treatment.

## Medical therapy

Medical therapy can be used short-term but is also suitable for long-term control. Side effects are specific for each drug (Table 4). Medical therapy is pituitary-directed (pasireotide and cabergoline), adrenal-directed (steroidogenesis inhibitors metyrapone, ketoconazole, mitotane and etomidate) and glucocorticoid-receptor directed (mifepristone), the last one currently not being approved in Europe. Efficacy of all drugs is quite variable; it is very high for steroidogenesis inhibitors and mifepristone, but lower for pasireotide and cabergoline [75]. Medical therapy can either be conducted as monotherapy or in combination with radiation therapy. Many drugs may be combined with each other; treatment can be performed as a block-and-replace-strategy or by titration of the drugs. Pituitary-directed medical therapy is reserved for CD while all the other drugs can be given in all forms of CS. Pasireotide is more effective in patients with mild or moderate hypercortisolism, with urinary free cortisol levels of up to two-times the normal range reaching control rates in ~40% [76]. Pasireotide treatment also leads to reduction of tumor volume [77]. Cabergoline is effective in up to 40% of patients, but 22% of patients escaped from remission over the long-term [78]. Adrenal steroidogenesis inhibitors block one or more enzymes in the steroidogenesis pathway. Glucocorticoid receptor blockers reduce the activity of cortisol on the glucocorticoid receptor. Advantages of medical therapy include the lower risk for adrenal insufficiency than by surgery and no risk for hypopituitarism. They are relatively safe to us if administered correctly. As their effect is immediate, they are the first choice in emergency situations with severe hypercortisolism. Medical therapy is also an option for patients who are no suitable candidates for surgery due to comorbidities. Disadvantages include the side effects of the medication, the high costs when used over a long-time period and the fact, that it is not a definite therapy. Furthermore, escape phenomena are possible (though seldom) leading to a recurrence of hypercortisolism.

**Table 4:** Medical therapy in CD [138, 139]

Drug	Application	Side effects	Further comments
Pasireotide	Subcutanous or intramuscular	Hyperglycemia, gall stones	Helpful in mild and moderate CD, not in severe CS
Cabergolin	Oral	Fatigue; compulsive behavior, addiction to games, sex addiction, heart valve disease, low blood pressure, etc	Helpful in mild and moderate CD, not in severe CS
Metyrapone	Oral	Hypokalemia, hirsutism, gastrointestinal, arrhythmia	Intake at least 3–4 times a day, can be combined with other drugs
Ketoconazole	Oral	Liver enzyme elevation, liver failure, arrhythmia, hypokaliaemia, interactions with other drugs	Can be combined with other drugs
Mifepristone	Oral	Nausea, fatigue, hypokaliaemia, peripheral oedema, endometrial thickening, abortifacient in early pregnancy	Dexamethasone as antagonist, not hydrocortisone no biochemical surveillance, only clinical

New adrenal steroidogenesis blockers and glucocorticoid receptor blockers such as osilodrostat [79], levoketoconazole [80], or relacorilant [81] are currently tested in phase II and phase III studies. There are also trials on new pituitary-directed drugs (for example retinoic acid) [82].

Although medical therapy can lower cortisol levels, according to a recent meta-analysis data regarding the clinical improvement or quality of life in patients treated with medical therapy are rare [75]. Also, it is unclear if medical therapy has different effects in patients with therapy-naïve CD or in patients with recurrent CD.

## Bilateral adrenalectomy

Bilateral adrenalectomy is considered as an emergency option in severe and uncontrolled hypercortisolism [83] or as a last option, when other treatment options are unsuccessful or not well-tolerated. Bilateral adrenalectomy is a definitive therapy with a success rate of 100%. In a recent metanalysis, it has been shown that besides being a safe treatment option with a surgical mortality of 3% [84], it also improves comorbidities and leads to an improvement of health-related quality of life [85]. The reasons why bilateral adrenalectomy is considered *ultima ratio*, are Nelson’s syndrome and permanent adrenal insufficiency. Nelson’s syndrome develops with a prevalence between 8 and 29% in studies with patient populations over 40 years [86]. Nelson’s syndrome, defined as cortisotroph tumor progression, is diagnosed by elevated plasma ACTH levels, hyperpigmentation, and tumor growth [87]. One study suggests that Nelson’s syndrome can be prevented by a prophylactic radiation therapy [88] but evidence for this approach is low, so it is not officially recommended. A hypophysectomy is seldom required [88]. The need for life-long hydrocortisone and fludrocortisone replacement therapy with the risk of having adrenal crises is the other main disadvantage of bilateral adrenalectomy.

## Treatment recommendations in biochemical and clinical mild recurrence

In patients with very mild hypercortisolism and no clinical signs of active CS, a wait and watch strategy can be practiced if there remain doubts on a recurrence. However, as known from patients with subclinical adrenal CS, cardiovascular outcome can be negatively influenced even by mild hypercortisolism [89]. Based on our clinical experience, we highly recommend to start treatment early in recurrent disease to improve both quality of life and long-term outcome.

Carroll et al. showed in a study with 15 patients that patients benefit from early initiation of second-line treatments in terms of weight-loss, improvement of hypertension and Hba1c, and improved quality of life, respectively. Of the 15 patients, 12 had normal UFC at time of recurrence, whereas the 1 mg dexamethasone suppression test was abnormal in 11 of 15, and 14 had abnormal late night salivary cortisol levels [90].

Keeping this in mind, we recommend to start treatment in these patients early, for example with low dose metyrapone, ketoconazole, or pasireotide in patients with negative MRI. Treatment with pasireotide might be a treatment option in patients with mild hypercortisolism [76] since success rate depends on the degree of hypercortisolism. In patients with a positive MRI, a second pituitary surgery should be considered early on. Comorbidities such as diabetes, arterial hypertension, and osteoporosis should always be treated adequately in parallel. Also, depression elevates the risk of cardiovascular diseases [91] and should be treated.

## Special remarks on recurrence of CD during pandemics

Due to an expert consensus, diagnosis and treatment of recurrent CD must not be delayed due to pandemic crisis such as the currently worldwide COVID-19-Crisis. On the other hand—as patients with CD are immunocompromised and at risk of infections—alternative consultation ways such as video and telephone might be used. Strict adherence to social distancing recommendations are highly recommended. Furthermore, medical treatment might be the first-line therapy in this scenario as treatment should not be delayed by extensive diagnostic procedures, however, hypercortisolism should be controlled as soon as possible [92].

## Overall recommendations: expertize and interdisciplinary tumor boards

Patients benefit from treatment by an experienced endocrinologist and neurosurgeon; the same applies for the diagnostic process and treatment decisions. As CD is a rare and complex disease, we highly recommend that patients should be treated in an experienced center that is specialized in CS. For treatment decisions, interdisciplinary tumor board have proven their worth.

## Summary and conclusion

Recurrence is a common problem in patients with CD occurring in around 15% of patients. The time to relapse varies between 3 and 158 months (mean 51 months), and 50% of relapses occur during the first 50 months after initial surgery. Annual clinical screening is recommended. In case of clinical and biochemical evidence of recurrence, co-morbidities should be immediately treated and the best option for secondary treatment should be selected. This decision requires a high level of expertize and transfer of the patient to a tertiary center is highly advised. If second TSS, radiation therapy or long-term medical therapy are no options, bilateral adrenalectomy is indicated with immediate control of hypercortisolism and improved long-term outcome.
